# Exploring the Cross-Sectional Association Between Hypothyroidism and Circadian Syndrome: Insights from NHANES 2007–2012

**DOI:** 10.3390/clockssleep7040052

**Published:** 2025-09-24

**Authors:** Ahmed Arabi, Humam Emad Rajha, Osama Alkeilani, Ahmad Hamdan, Dima Nasrallah, Giridhara R. Babu

**Affiliations:** 1 College of Medicine, Qatar University (QU) Health, Qatar University, Doha P.O. Box 2713, Qatar; as2106002@qu.edu.qa (A.A.); hr2003606@qu.edu.qa (H.E.R.); oa2103855@qu.edu.qa (O.A.); ah1904442@qu.edu.qa (A.H.); dn2105571@qu.edu.qa (D.N.); 2 Department of Population Medicine, College of Medicine, Qatar University (QU) Health, Qatar University, Doha P.O. Box 2713, Qatar

**Keywords:** hypothyroidism, thyroid dysfunction, circadian syndrome (CircS), metabolic syndrome (MetS), type 2 diabetes, cardiovascular disease, NHANES

## Abstract

Background: Circadian Syndrome (CircS) encompasses a range of cardiometabolic risk factors that contribute to an increased susceptibility to cardiovascular diseases and type 2 diabetes. Understanding the factors that underpin CircS is essential. This study primarily aims to examine the association between hypothyroidism and CircS in adults. A secondary analysis compares this association with that between hypothyroidism and Metabolic Syndrome (MetS). Additionally, the dose–response relationship between serum free thyroxine (FT4) levels and CircS probability is explored. Methods: This cross-sectional study includes 4050 National Health and Nutrition Examination Survey (NHANES) participants (2007–2012). Hypothyroidism was classified into (1) drug-managed, (2) non-drug-managed (NDM) primary, and (3) NDM central hypothyroidism, based on self-reported medication use and serum TSH/FT4 levels. CircS was defined as having ≥5 of its eight components, including MetS criteria, depression, short sleep, and non-alcoholic fatty liver disease. Results: Our results showed that hypothyroidism was significantly associated with CircS (OR: 1.58, 95% CI 1.26–1.98) and MetS (OR: 1.19, 95% CI 1.01–1.42). An inverse, non-linear relationship between serum FT4 levels and the probability of CircS was observed. Conclusions: The results underscore a significant association between hypothyroidism and CircS and MetS, with FT4 levels inversely related to CircS probability. These findings highlight hypothyroidism’s potential role in CircS pathogenesis and prevention.

## 1. Introduction

Circadian syndrome (CircS) is an emerging, holistic concept that unifies a cluster of cardiometabolic risk factors and associated comorbidities, reflecting the risk of developing cardiovascular diseases (CVDs) and type 2 diabetes mellitus (T2DM) [1]. CircS expands on the foundation of metabolic syndrome (MetS), which encompasses central obesity, elevated fasting plasma glucose (FPG), dyslipidemia defined by high triglycerides (TG) and/or low high-density lipoprotein (HDL) cholesterol, and elevated blood pressure [2]. Notably, CircS incorporates three additional components, including sleep disturbances, depression, and non-alcoholic fatty liver disease (NAFLD) [1]. A key distinctive feature of CircS, compared to MetS, is its etiological foundation, as all eight components are unified by an underlying disruption of the circadian rhythm [1]. Circadian rhythm, regulated by the hypothalamic suprachiasmatic nucleus, commonly referred to as the master circadian clock, orchestrates various key physiological processes, including hormonal release, gene expression, activity patterns, and energy expenditure [3]. Disruptions in this tightly regulated circadian rhythm result in malfunctioning in almost all metabolic processes in the body, which is at the core of CircS development [4].

Given CircS’s clear etiological base and well-established association with CVDs and T2DM, identifying and mitigating factors contributing to circadian clock disruption are essential. Such interventions can potentially prevent or reverse CircS, alleviating the associated burden of CVDs and T2DM. Recent research has shed light on the role of thyroid hormones as potential regulators of circadian rhythm genes [5]. Hypothyroidism has been found to disrupt the transcription of *Bmal1*, a core circadian rhythm gene, causing decreased neuronal activity in the suprachiasmatic nucleus and ultimately disrupting the circadian rhythm. Additionally, disruption of *Bmal1* expression has been implicated in decoupling the master circadian clock from peripheral circadian clocks located in vital organs, thereby altering functionality and disrupting rhythmicity across different organ systems in the body [5].

Notably, the expression of circadian rhythm genes in the peripheral clocks of cardiomyocytes is impacted explicitly in hypothyroidism, leading to the loss of diurnal rhythmicity of cardiac metabolic processes. This increases the risk of myocardial damage and metabolic stress, which are precursors to CVDs [6]. Additionally, hypothyroidism has been implicated in insulin resistance, a cornerstone in both MetS and CircS, by downregulating the circadian-controlled expression of proinsulin genes, further reinforcing its link to cardiometabolic disturbances [7,8,9]. Furthermore, hypothyroidism is associated with depression [10], NAFLD [11], and sleep disturbances [12], constituting the additional components of CircS. Hence, hypothyroidism is postulated to not only increase the risk of CVDs directly but also contribute to the development of CircS, itself a significant risk factor of CVDs.

Given the limited evidence in existing literature regarding the association between hypothyroidism and CircS, this study aims to explore the association between hypothyroidism status and circadian syndrome status among adults enrolled in the National Health and Nutrition Examination Survey (NHANES) in the period spanning from 2007 to 2012 as a primary outcome. A secondary objective was to compare this association with the relationship between hypothyroidism status and MetS status. Furthermore, the dose–response relationship between serum free thyroxine (FT4) levels and the probability of CircS was examined to understand the interplay between thyroid function and CircS.

## 2. Results

### 2.1. Baseline Characteristics

A total of 4050 participants were included in the primary analysis of this study, of whom 12% (n = 474) were identified as having circadian syndrome. The baseline characteristics of the study population are detailed in Table 1. Participants with CircS were older, with a median age of 57 years, compared to 44 years among those without CircS. Both groups were predominantly composed of white individuals, and a PIR of 1–1.9 was the most common class across both groups. Individuals in the CircS group exhibited a higher prevalence of poor diet quality, vitamin D deficiency, chronic kidney disease, and liver cirrhosis. In contrast, those in the no CircS group demonstrated higher physical activity levels. Notably, hypothyroidism was more common among participants with CircS compared to those without (31.6% vs. 18.5%, respectively).

### 2.2. Hypothyroidism Prevalence

**Figure 1:**
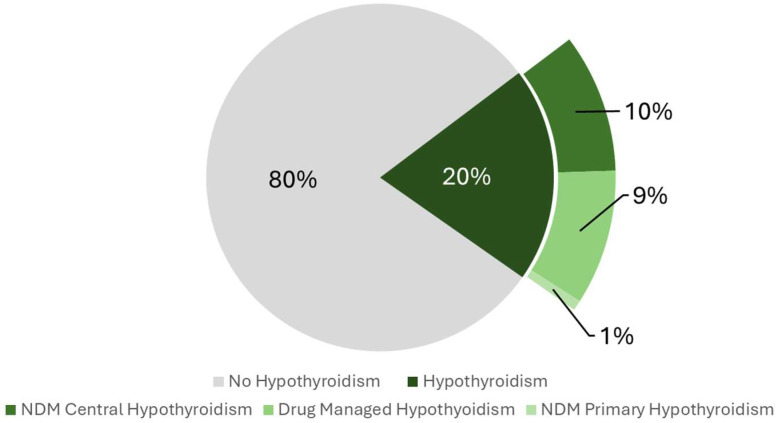
Pie Chart illustrating the prevalence of hypothyroidism and its subtypes among participants included in the study (n = 4050). NDM: Non-Drug-Managed.

Figure 1 illustrates the unweighted prevalence of hypothyroidism and its various subtypes among the study participants. Among the 4050 individuals included in the primary outcome analysis, 20% (n = 810) were identified as having hypothyroidism. The most prevalent subtype was NDM central hypothyroidism, accounting for 10% of cases, followed closely by drug-managed hypothyroidism, which accounted for 9%. The least common subtype observed was NDM primary hypothyroidism, representing just 1% of cases.

### 2.3. Association of Hypothyroidism Status with CircS and MetS

The association between hypothyroidism status and CircS and MetS was assessed through multivariable logistic regression analyses, with the adjusted ORs presented in Table 2 and Table 3, respectively, and unadjusted ORs presented in Tables S1 and S2. The adjusted findings demonstrate that individuals with any form of hypothyroidism exhibited 58% higher odds of having CircS compared to those without hypothyroidism (OR: 1.58, 95% CI 1.26–1.98, *p* < 0.001), as reported in Table 2. Additionally, as shown in Table 3, participants with hypothyroidism experienced a 1.19-fold increase in the odds of having MetS compared to their counterparts without hypothyroidism (OR: 1.19, 95% CI 1.01–1.42, *p* = 0.042). To investigate the potential sex-based differences in the context of the higher population prevalence of hypothyroidism among women, we employed sex-stratified, covariate-adjusted analyses to examine the associations between hypothyroidism and both CircS and MetS in Tables S3 and S4.

### 2.4. Dose–Response Relationship Between Serum Free Thyroxine and CircS Probability

**Figure 2:**
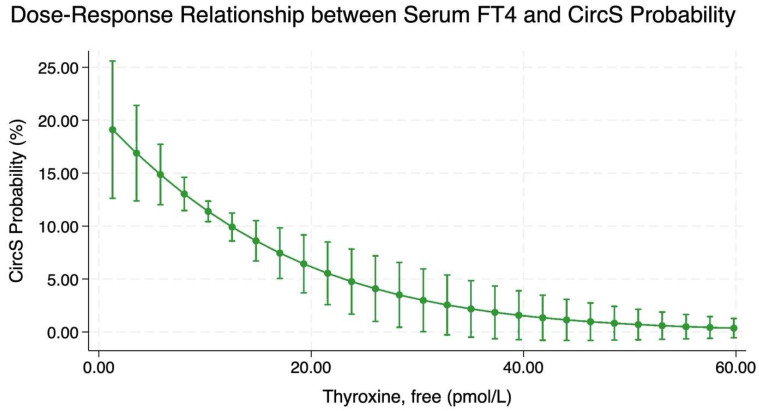
Dose–response curve demonstrating the association between serum FT4 and CircS probability (n = 3878). Model adjusted for age (centered at the mean value of 47.2 years), sex, race, poverty income ratio, education level, diet quality, physical activity (centered at the mean value of 65.5 MET hours per week), vitamin D status, CKD, and liver cirrhosis.

Figure 2 and Figure S2 depict the adjusted and unadjusted dose–response relationship between serum FT4 levels and the probability of CircS, respectively. The adjusted results indicate that an inverse, non-linear association was observed, with higher serum FT4 levels corresponding to a reduced predicted probability of CircS. Specifically, participants with a baseline serum FT4 level of 1.3 pmol/L exhibited a 19% (95% CI: 12–25%) probability of CircS. In contrast, those with a baseline serum FT4 level of 12.5 pmol/L had a 10% (95% CI: 8.5–11.2%) CircS predicted probability. The probability of CircS continues to decline as serum FT4 levels increase, up to approximately 40 pmol/L, beyond which the decline plateaus, suggesting minimal to no further reduction in CircS probability with additional increases in serum FT4. The adjusted dose–response relationship between serum FT4 levels and the probability of MetS are presented in Figure S3. The adjusted results indicate that an inverse, non-linear association was observed, with higher serum FT4 levels corresponding to a reduced predicted probability of CircS. Conversely, the dose–response relationship between serum TSH levels and the probability of CircS showed a direct association (Figure S4).

## 3. Discussion

This study explored the association between hypothyroidism and important cardiometabolic risk determinants, namely CircS and MetS, using a large representative sample from NHANES. Compared to individuals with no hypothyroidism, those with hypothyroidism exhibited a 58% increase in the adjusted odds of CircS and a 19% increase in the adjusted odds of metabolic syndrome (MetS). A non-linear inverse relationship was observed between serum FT4 levels and CircS probability when adjusting for covariates, with lower serum FT4 levels correlating with higher CircS probabilities until it plateaus at around a serum FT4 level of 40 pmol/L.

Given that the evidence about CircS is emerging, research linking it to hypothyroidism remains sparse. However, the association between hypothyroidism and MetS is well-documented in the literature. Consistent with prior studies, our findings support the role of hypothyroidism as a potential risk factor for MetS and its different components [13,14,15,16]. Further corroborating our results, meta-analyses by Yang et al. (2016) and Ding et al. (2021) emphasize the link between subclinical hypothyroidism and MetS [17,18].

Our study, for the first time, demonstrates that, beyond the five components of MetS, hypothyroidism is also associated with three additional components of CircS. While previous research has provided evidence for these three components individually, their combined association within CircS has not been explored until now. For instance, it is well-established that hypothyroidism significantly increases the risk of depression, as reported in a meta-analysis by Bode et al. (2021), which found a 1.77-fold increase in the odds of depression among individuals with hypothyroidism (95% CI 1.13–2.77) [19]. The relationship between hypothyroidism and NAFLD, however, remains contentious. While some studies report no significant association [20,21], others have identified a strong link, particularly in the context of systemic metabolic dysfunction [22,23]. Proposed mechanisms include hypothyroidism-induced insulin resistance, dyslipidemia, and obesity, which, in turn, exacerbate hepatic injury [24]. Additionally, hypothyroidism impairs lipid metabolism, increasing cholesterol, low-density lipoprotein (LDL), and triglycerides while reducing HDL, thereby promoting hepatic fat accumulation [25]. Finally, the last component of CircS, sleep disturbance, which underscores a significant influence on cardiometabolic health, has been linked to thyroid dysfunction, particularly hypothyroidism. This association was observed among various types of sleep disturbances, including, but not limited to, insomnia, restless leg syndrome, and obstructive sleep apnea [26]. Further supporting this, subclinical hypothyroidism has also been associated with sleep disturbances. A systematic review by Teliti et al. (2024) reported a positive correlation between subclinical hypothyroidism and sleep disturbances in seven out of eight studies included [27].

Our findings reveal a robust association between hypothyroidism and CircS, evidenced by a 1.58-fold increase in the odds of CircS among adults with hypothyroidism compared to those without hypothyroidism, alongside an inverse non-linear dose–response relationship between serum FT4 levels and CircS probability. These findings underscore the central role of thyroid hormones in regulating the circadian rhythm, which in turn controls critical biological processes, potentially serving as one of the key mechanisms underlying homeostasis of metabolic functions. The suprachiasmatic nucleus, functioning as the molecular circadian clock, governs metabolic processes by synchronizing the expression of critical enzymes and hormones throughout the diurnal cycle [5]. This involves the expression of key circadian proteins, including *Clock* and *Bmal1*, which activate genes responsible for glucose metabolism, lipid synthesis, and energy production, ensuring alignment of these processes with periods of activity and rest [6,28,29,30]. Intriguingly, the expression of these genes is regulated by various metabolites and hormones, with thyroid hormones standing out as significant regulators of their expression [5].

However, in hypothyroidism, the expression of these circadian clock proteins is altered, resulting in disturbances in the circadian rhythm and, consequently, significant disruptions in major metabolic processes [5,6,31]. In thyroidectomy rat models, reduced thyroid hormone levels were linked to altered Per2 expression in the amygdala and bed nucleus of the stria terminalis, providing insights into how thyroid dysfunction alters the circadian cycle [32,33]. These findings align with our results, reinforcing that thyroid dysfunction is critical in circadian system dysregulation and its downstream effects on cardiometabolic risk indicators. Therefore, from a broader perspective, circadian rhythm disruption is proposed to be accountable for the various components of CircS, suggesting that CircS provides a more comprehensive reflection of cardiometabolic health than MetS. This is further supported by the stronger association observed in our study between hypothyroidism and CircS compared to MetS. Taken together, this highlights the significance of thyroid function in maintaining circadian homeostasis and mitigating associated cardiometabolic complications.

To the best of our knowledge, this study is the first to not only explore the association between hypothyroidism and circadian syndrome but also the dose–response relationship between serum FT4 levels and the probability of circadian syndrome, establishing thyroid hormone levels as a key factor in regulating the circadian cycle. A notable strength of this research is the utilization of the NHANES database, renowned for its representativeness of the general U.S. population. Furthermore, including a substantial sample size comprising 4050 participants enhances the generalizability of our findings, albeit primarily within the context of the U.S. population.

Despite these strengths, the study has several limitations. First, the cross-sectional design of NHANES precludes inferences regarding causality, directionality, or temporality. Further, there is an interdependence between circadian rhythm regulation and the endocrine system, particularly concerning thyroid-stimulating hormone (TSH), which is itself regulated by the circadian system [34], complicating inference. Disruptions in circadian rhythms, including night shift work, have been implicated in thyroid dysfunction [34]. The possibility of a bidirectional relationship between hypothyroidism and circadian rhythm disturbances necessitates conducting longitudinal research to establish temporality. It is important to note that sleep duration is only one aspect of the circadian system’s regulation of the sleep–wake cycle. Sleep quality and sleep disorders are also significant components of circadian rhythm disruption. However, due to limitations in the available data and based on previous literature, we made the decision to represent the sleep–wake cycle primarily through sleep duration in this study. Future research with more comprehensive data could explore the full range of circadian factors, including sleep quality and disorders, to better capture the complexity of circadian system regulation. Reliance on certain self-reported variables and using proxies to replace the absence of specific variables (such as the US-FLI and APRI for diagnosing NAFLD and cirrhosis, respectively) are limitations about the NHANES dataset.

The findings of this study carry significant implications for both clinical practice and public health, emphasizing the need for further investigations of hypothyroidism and circadian syndrome. Given the strong association between hypothyroidism and circadian rhythm disruption, clinicians can consider evaluating circadian health in patients with thyroid disorders, including monitoring sleep patterns, metabolic risk factors, and mental health. Furthermore, our results highlight the potential utility of targeting circadian rhythms as part of comprehensive management strategies for hypothyroidism, particularly in mitigating its cardiometabolic and psychological impacts. Interventions such as tailored hormone replacement therapy, lifestyle modifications, and strategies to enhance circadian alignment, such as exposure to natural light and optimized meal timing, could prove beneficial. Longitudinal studies are imperative to establish causality and assess the long-term effects of hypothyroidism on the development and progression of CircS. Future research could also explore therapeutic options to restore circadian clock gene function to enhance overall health outcomes in individuals with thyroid dysfunction.

## 4. Materials and Methods

### 4.1. Study Design and Sample

Study method and results are reported following the Strengthening the Reporting of Observational Studies in Epidemiology (STROBE) Statement for cross-sectional studies [35]. The National Health and Nutrition Examination Survey (NHANES), administered by the Centers for Disease Control and Prevention (CDC), is a nationally representative cross-sectional survey. It is designed to evaluate the health and nutritional status of the United States population. NHANES employs a multistage probability sampling methodology to ensure inclusivity and demographic representation across all 50 states. The survey integrates a multifaceted approach to data collection, encompassing structured face-to-face or telephonic interviews, comprehensive questionnaires, laboratory analyses, and detailed physical examinations [36]. Ethical governance is rigorously upheld through the oversight of the Institutional Review Board of the National Center for Health Statistics, with informed written consent obtained from all participants prior to their involvement. The survey’s data and methodologies are publicly available at https://www.cdc.gov/nchs/nhanes/?CDC_AAref_Val=https://www.cdc.gov/nchs/nhanes/index.htm (accessed on 26 December 2024).

We used data from three NHANES cycles conducted between 2007 and 2012, comprising 30,442 participants. For the primary outcome analysis, we excluded individuals aged <18 years (n = 11,823), those with a self-reported history of malignancy (n = 1684), participants with incomplete data on hypothyroidism status (n = 8199) or CircS status (n = 2762), and those with incomplete data on other covariates (n =1924). Following these exclusions, the final analytical sample for the primary outcome analysis included 4050 participants. As for the secondary outcome analysis, which examined the association between hypothyroidism status and MetS status, a final subset of 4022 participants aged ≥18 years who had no self-reported history of malignancy and complete data on hypothyroidism status was utilized after excluding those with incomplete data on MetS status or other covariates. Lastly, in the analysis investigating the dose–response relationship between serum FT4 levels and the probability of CircS, the subset of 16,935 participants aged ≥18 years with no self-reported history of malignancy was utilized. Participants were excluded if they had incomplete data on serum FT4 levels (n = 8566), CircS status (n = 2606), or other covariates (n = 1885), yielding a final analytical sample of 3878 participants.

### 4.2. Exposure Measure: Hypothyroidism Status

In this study, hypothyroidism was characterized by the presence of any of the following three conditions: (1) Drug-managed hypothyroidism, (2) non-drug-managed (NDM) primary hypothyroidism, or (3) NDM central hypothyroidism. Drug-managed hypothyroidism was defined as the self-reported use of thyroid hormone replacement therapy within the past month. NDM primary and central hypothyroidism were determined based on serum levels of TSH and FT4 in participants who reported no use of thyroid hormone replacement therapy or antithyroid medications. According to the laboratory procedure manuals [37], the reference ranges for TSH and FT4 were 0.34–5.6 mIU/L and 7.74–20.6 pmol/L, respectively. TSH levels exceeding 5.6 mIU/L in conjunction with an FT4 level below 7.74 pmol/L were indicative of NDM primary hypothyroidism. As for NDM central hypothyroidism, either TSH within the normal reference range with FT4 below 7.74 pmol/L or TSH below 0.34 mIU/L with FT4 below 7.74 pmol/L were suggestive [37].

### 4.3. Outcome Measure: Circadian Syndrome Status

CircS was assessed based on eight components, with a diagnostic threshold of ≥5 components. These components include: (1) depression, as indicated by a Patient Health Questionnaire (PHQ-9) score of ≥10/27 [38]; (2) short sleep duration, defined as self-reported sleep of less than 6 h per day [39]; and (3) non-alcoholic fatty liver disease (NAFLD). The remaining five components correspond to the criteria for MetS [2,40], encompassing: (4) elevated waist circumference (≥102 cm for males and ≥88 cm for females); (5) elevated blood pressure (systolic ≥130 mm Hg and/or diastolic ≥85 mm Hg) or the use of antihypertensive medications in patients with hypertension; (6) low high-density lipoprotein (HDL) cholesterol levels (<40 mg/dL for men and <50 mg/dL for women) or treatment for reduced HDL cholesterol; (7) high triglycerides (≥150 mg/dL) or treatment for elevated triglycerides; and (8) elevated fasting plasma glucose (FPG) (≥100 mg/dL), or treatment for elevated glucose, or a diagnosis of diabetes or prediabetes.

NAFLD status was determined using the United States Fatty Liver Index (US-FLI). NAFLD was defined as a US-FLI score of ≥30, provided there were no other etiologies for liver disease, such as excessive alcohol consumption (more than four standard drinks per day for males and more than three standard drinks per day for females [41]) or hepatitis B infection (HBsAg positive).

### 4.4. Covariates

The following covariates, including confounders and prognostic factors, were included: demographics, lifestyle, chronic diseases, and serum vitamin D status variables. Demographic variables comprised self-reported age (in years), gender, race (Hispanic, White, Black, or others/multiracial), and socioeconomic status, which was determined using educational level (below high school, high school, and graduate) and poverty income ratio (PIR) categories (<1, 1–1.9, 2–2.9, 3–3.9, 4–4.9, and ≥5 [42]). Lifestyle variables included dietary quality and physical activity. Dietary quality was assessed using the Healthy Eating Index-2020 (HEI-2020), which evaluates adherence to the 2020–2025 Dietary Guidelines for Americans through 13 components [43]. Participants with HEI-2020 scores in or above the 60th percentile were classified as having high dietary quality [44]. Physical activity was evaluated using the Global Physical Activity Questionnaire (GPAQ), covering vigorous- and moderate-intensity work activities, walking or bicycling for transportation, and vigorous- and moderate-intensity leisure activities [45]. Activities were assigned respective Metabolic Equivalent of Task (MET) scores, and total MET hours per week were aggregated to represent physical activity levels, with higher values indicating greater activity. Chronic disease variables included self-reported chronic kidney disease (CKD) status and liver cirrhosis; the latter identified using an Aspartate Aminotransferase (AST) to Platelet Ratio Index (APRI) score >1 [46]. Lastly, serum vitamin D status was defined as the combined concentrations of 25(OH)D3 and 25(OH)D and categorized into adequate (≥50 nmol/L or ≥20 ng/mL), inadequate (30 to <50 nmol/L or 12 to <20 ng/mL), and deficient (<30 nmol/L or <12 ng/mL) levels, following the Institute of Medicine reference ranges [47].

### 4.5. Statistical Analysis

Categorical variables were reported as frequencies (N) and proportions (%). Continuous variables underwent an initial assessment of normality through histograms, which revealed a non-normal distribution for all variables. Consequently, they were summarized using medians and interquartile ranges (IQR). Group comparisons were performed utilizing Pearson’s chi-squared test for categorical variables and the Wilcoxon rank-sum test for continuous variables. Odds ratios (OR) were generated using multivariable logistic regression models to examine the association between each exposure and outcome while adjusting for covariates, whether prognostic factors or confounders, identified via Directed Acyclic Graphs (DAG) (Supplementary Figure S1). The dose–response relationship between serum FT4 levels and the probability of CircS was visualized using Stata’s *marginsplot* function, centering age and physical activity on their mean values. Goodness of fit was evaluated through the Receiver Operating Characteristic (ROC) curve and the corresponding Area Under the Curve (AUC), while the *linktest* in Stata was applied to assess the model’s goodness of link. Confidence intervals (95% CI) and *p*-Values were provided where applicable. Data visualization was performed using pie charts and line graphs as appropriate. All statistical analyses were conducted using Stata version SE18.5 (Stata Corp., College Station, TX, USA).

## 5. Conclusions

Our findings demonstrate a significant association between hypothyroidism and key cardiometabolic risk factors, specifically Circadian Syndrome (CircS) and Metabolic Syndrome (MetS). When comparing the relationship between hypothyroidism and MetS to that with CircS, it is evident that the association with CircS is substantially stronger. The observed dose–response relationship reveals an inverse, non-linear pattern, wherein lower serum free thyroxine levels are associated with an increased probability of CircS. This study underscores the potential public health benefits of early detection and management of hypothyroidism to mitigate its effects on CircS and, by extension, cardiometabolic health. Future longitudinal studies are needed to establish the causal role of hypothyroidism in CircS and assess thyroid replacement therapy’s effectiveness in preventing circadian dysfunction.

## Figures and Tables

**Table 1:** Baseline characteristics of the study’s population categorized by CircS status (n = 4050).

Characteristics	Categories	No Circadian Syndrome (n = 3576)	Circadian Syndrome (n = 474)	*p*-Value
**Age in years, Median (IQR)**		44.00 (31.00, 60.00)	57.00 (46.00, 67.00)	<0.001 ^1^
**Sex**				
	Male	1795 (50.2%)	208 (43.9%)	0.01 ^2^
	Female	1781 (49.8%)	266 (56.1%)	
**Race**				
	Hispanic	922 (25.8%)	133 (28.1%)	<0.001 ^2^
	White	1683 (47.1%)	256 (54.0%)	
	Black	695 (19.4%)	73 (15.4%)	
	Others/multi-racial	276 (7.7%)	12 (2.5%)	
**Poverty Income Ratio (PIR)**				
	PIR < 1	686 (19.2%)	126 (26.6%)	<0.001 ^2^
	PIR 1–1.9	922 (25.8%)	160 (33.8%)	
	PIR 2–2.9	582 (16.3%)	68 (14.3%)	
	PIR 3–3.9	406 (11.4%)	38 (8.0%)	
	PIR 4–4.9	312 (8.7%)	27 (5.7%)	
	PIR ≥ 5	668 (18.7%)	55 (11.6%)	
**Education Level**				
	Below high school	844 (23.6%)	164 (34.6%)	<0.001 ^2^
	Highschool	1806 (50.5%)	249 (52.5%)	
	Graduate	926 (25.9%)	61 (12.9%)	
**Serum Vitamin D Level**				
	Adequacy	2464 (68.9%)	321 (67.7%)	0.45 ^2^
	Inadequacy	812 (22.7%)	105 (22.2%)	
	Deficiency	300 (8.4%)	48 (10.1%)	
**Diet Quality**				
	Low Dietary Quality	2011 (56.2%)	282 (59.5%)	0.18 ^2^
	High Dietary Quality	1565 (43.8%)	192 (40.5%)	
**Physical Activity Status, Median (IQR)**		22.00 (3.33, 80.00)	8.00 (0.00, 44.00)	<0.001 ^1^
**Chronic Kidney Disease Status**				
	No CKD	3513 (98.2%)	444 (93.7%)	<0.001 ^2^
	CKD	63 (1.8%)	30 (6.3%)	
**Liver Cirrhosis Status**				
	No Cirrhosis	3523 (98.5%)	461 (97.3%)	0.042 ^2^
	Cirrhosis	53 (1.5%)	13 (2.7%)	
**General Hypothyroidism status**				
	No Hypothyroidism	2916 (81.5%)	324 (68.4%)	<0.001 ^2^
	General Hypothyroidism	660 (18.5%)	150 (31.6%)	

^1^ *p*-value generated using Wilcoxon rank-sum. ^2^ *p*-value generated using Pearsons’s chi-squared.

**Table 2:** Association between hypothyroidism status and CircS status among included participants (n = 4050) ^1^.

Exposure	Categories	CircS OR	*p*-Value	95% CI
**Hypothyroidism Status**				
	No Hypothyroidism	1		
	Hypothyroidism	1.58	<0.001	1.26–1.98

^1^ Model adjusted for age, sex, race, poverty income ratio, education level, diet quality, physical activity, vitamin D status, CKD, and liver cirrhosis.

**Table 3:** Association between hypothyroidism status and MetS status among included participants (n = 4022) ^1^.

Exposure	Categories	MetS OR	*p*-Value	95% CI
**Hypothyroidism Status**				
	No Hypothyroidism	1		
	Hypothyroidism	1.19	0.042	1.01–1.42

^1^ Model adjusted for age, sex, race, poverty income ratio, education level, diet quality, physical activity, vitamin D status, CKD, and liver cirrhosis.

## Data Availability

Publicly available datasets were analyzed in this study. The dataset presented in this study can be found at https://www.cdc.gov/nchs/nhanes/?CDC_AAref_Val=https://www.cdc.gov/nchs/nhanes/index.htm (accessed on 26 December 2024).

## Supplementary Materials

The following supporting information can be downloaded at: https://www.mdpi.com/article/10.3390/clockssleep7040052/s1, Figure S1: Directed Acyclic Graph (DAG) showing the association between hypothyroidism status with circadian syndrome and different covariates. Figure S2: Dose–response curve demonstrating the unadjusted association between serum FT4 and CircS probability. Figure S3: Dose–response curve demonstrating the adjusted association between serum TSH and MetS probability. Figure S4: Dose–response curve demonstrating the adjusted association between serum TSH and CircS probability. Table S1: Unadjusted association between hypothyroidism status and CircS status. Table S2: Unadjusted association between hypothyroidism status and MetS status. Table S3: Sex-stratified, covariate-adjusted association between hypothyroidism status and CircS status. Table S4: Sex-stratified, covariate-adjusted association between hypothyroidism status and MetS status.

## Abbreviations

The following abbreviations are used in this manuscript:

CircS	Circadian Syndrome
MetS	Metabolic Syndrome
FT4	Free Thyroxine
TSH	Thyroid-Stimulating Hormone
NHANES	National Health and Nutrition Examination Survey
NDM	Non-Drug Managed
CVDs	Cardiovascular Diseases
T2DM	Type 2 Diabetes Mellitus
TG	Triglycerides
HDL	High-Density Lipoprotein
NAFLD	Non-Alcoholic Fatty Liver Disease
SCN	Suprachiasmatic Nucleus
CKD	Chronic Kidney Disease
AST	Aspartate Aminotransferase
APRI	Aspartate Aminotransferase to Platelet Ratio Index
PHQ-9	Patient Health Questionnaire-9
FPG	Fasting Plasma Glucose
US-FLI	United States Fatty Liver Index
ROC	Receiver Operating Characteristic
AUC	Area Under the Curve
MET	Metabolic Equivalent of Task
DAG	Directed Acyclic Graph
HEI-2020	Healthy Eating Index-2020
PIR	Poverty Income Ratio
STROBE	Strengthening the Reporting of Observational Studies in Epidemiology
CDC	Centers for Disease Control and Prevention
