# Supplementary material for: Exploring the Cross-Sectional Association Between Hypothyroidism and Circadian Syndrome: Insights from NHANES 2007–2012
Source: Clocks Sleep. 2025 Sep 24;7(4):52. doi: 10.3390/clockssleep7040052 (PMC12550944; doi:10.3390/clockssleep7040052)
Supplement: Supplementary file 1 [file clockssleep-07-00052-s001.zip › clockssleep-3842185-supplementary.pdf]

Supplementary Material

Exploring the Association between Hypothyroidism and Circadian Syndrome: Insights from NHANES 2007-2012

Ahmed Arabi [1], Humam Emad Rajha [1], Osama Alkeilani [1], Ahmad Hamdan [1], Dima Nasrallah [1], Giridhara R. Babu [2,*]

[1] College of Medicine, QU Health, Qatar University, Doha P.O. Box 2713, Qatar

[2] Department of Population Medicine, College of Medicine, QU Health, Qatar University, Doha P.O. Box 2713, Qatar

[*] Corresponding author. E-mail address: gbabu@qu.edu.qa (G.R. Babu)

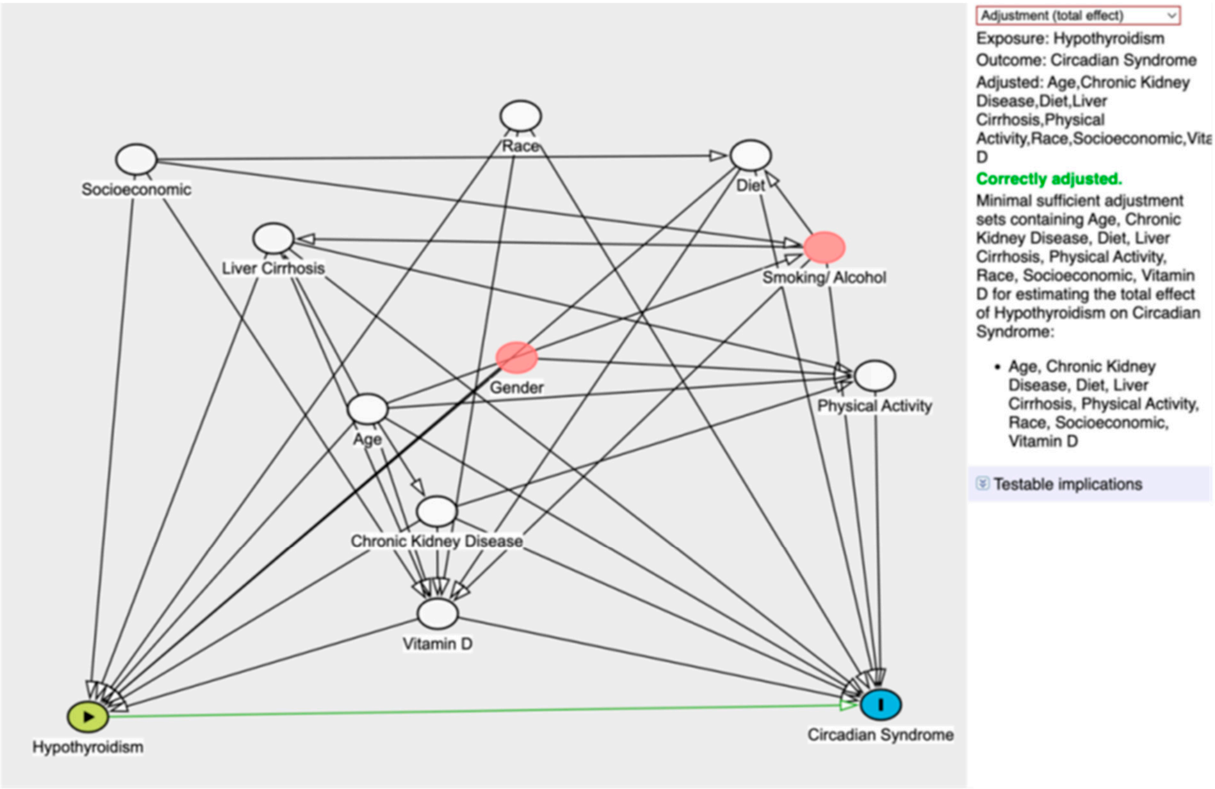

Figure S1. Directed Acyclic Graph (DAG) showing the association between hypothyroidism status with circadian syndrome and different covariates.

Table S1. Unadjusted association between hypothyroidism and CircS status (n = 5,974) [1].

Exposure	Categories	CircS OR	*p*-Value	95% CI
**Hypothyroidism Status**				
	No Hypothyroidism	1		
	Hypothyroidism	2.04	<0.001	1.69–2.45

[1] Included cohort for the unadjusted association includes adults (aged 18 and above) with complete data on hypothyroidism status and CircS status.

Table S2. Unadjusted association between hypothyroidism and MetS status (n = 5,935) [1].

Exposure	Categories	MetS OR	*p*-Value	95% CI
**Hypothyroidism Status**				
	No Hypothyroidism	1		
	Hypothyroidism	1.88	<0.001	1.65–2.15

[1] Included cohort for the unadjusted association includes adults (aged 18 and above) with complete data on hypothyroidism status and CircS status.

Table S3. Sex-stratified adjusted association between hypothyroidism and CircS status (n = 4,050) [1].

Exposure		Categories	CircS OR	*p*-Value	95% CI
**Hypothyroidism Status**					
**Male**	N = 2,003	No Hypothyroidism	1		
		Hypothyroidism	1.64	0.007	1.14–2.34
**Female**	N = 2,047	No Hypothyroidism	1		
		Hypothyroidism	1.70	<0.001	1.26–2.30

[1] Model adjusted for age, race, poverty income ratio, education level, diet quality, physical activity, vitamin D status, CKD, and liver cirrhosis.

Table S4. Sex-stratified adjusted association between hypothyroidism and MetS status (n = 4,022) [1].

**Exposure**		**Categories**	**MetS OR**	***p*-Value**	**95% CI**
**Hypothyroidism Status**					
**Male**	N = 1,930	No Hypothyroidism	1		
		Hypothyroidism	1.23	0.145	0.93–1.62
**Female**	N = 2,092	No Hypothyroidism	1		
		Hypothyroidism	1.25	0.054	1.00–1.57

[1] Model adjusted for age, race, poverty income ratio, education level, diet quality, physical activity, vitamin D status, CKD, and liver cirrhosis.

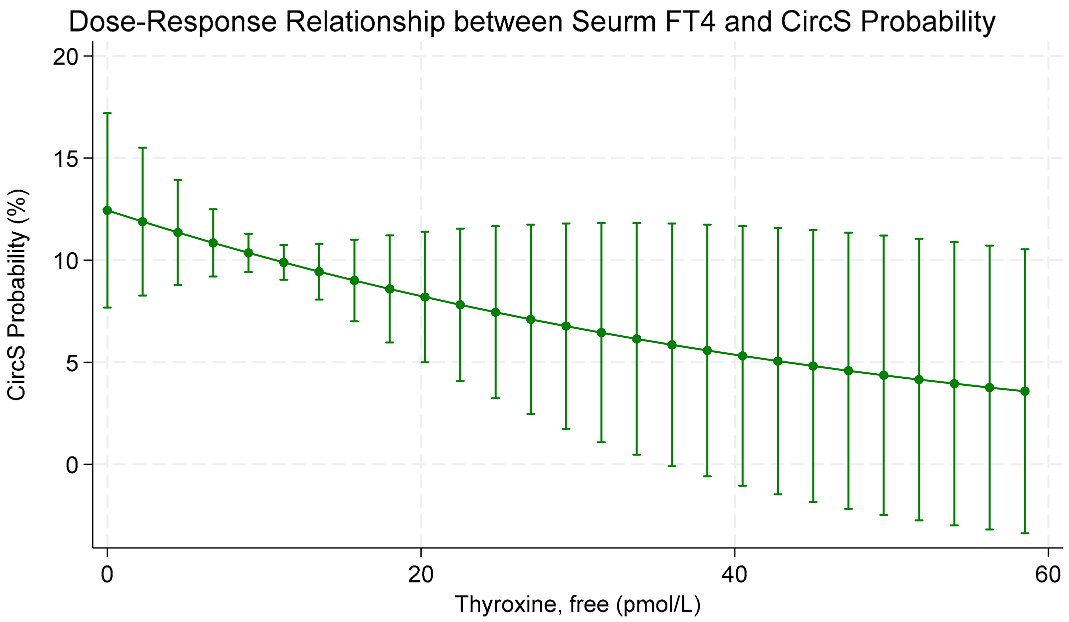

Figure S2. Dose-response Curve demonstrating the unadjusted association between serum FT4 and CircS probability (n= 5,763).[1]

[1] Included cohort for the unadjusted dose-response analysis includes adults (aged 18 and above) with complete data on serum FT4 and CircS status.

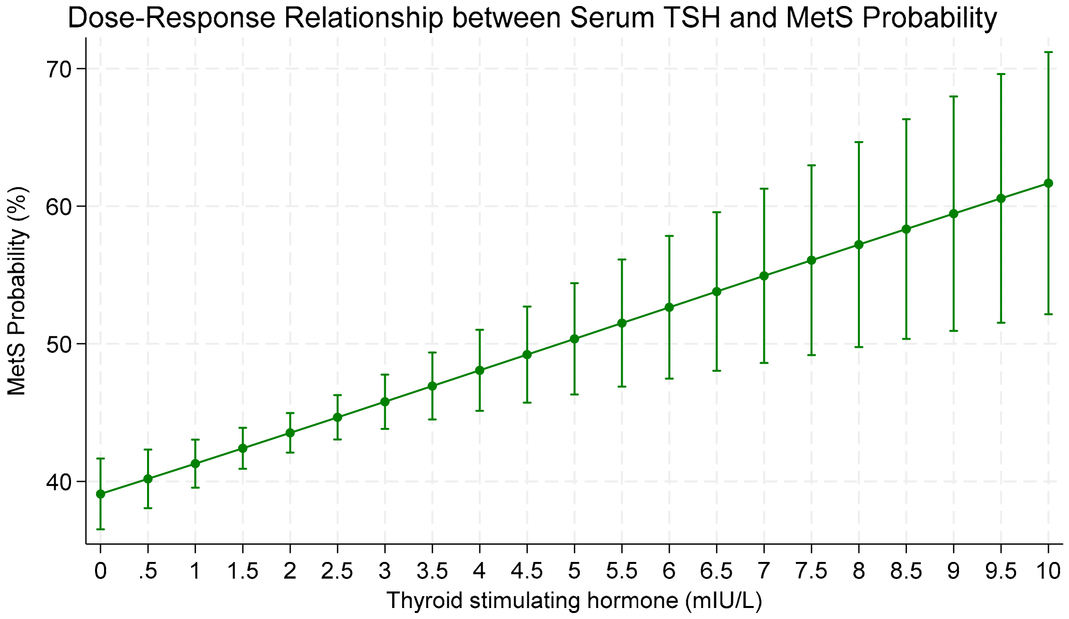

Figure S3. Dose-response Curve demonstrating the adjusted association between serum TSH and MetS probability (n= 3,853).[1]

[1] Model adjusted for age (centered at the mean value of 47.2 years), sex, race, poverty income ratio, education level, diet quality, physical activity (centered at the mean value of 65.5 MET hours per week), vitamin D status, CKD, and liver cirrhosis.

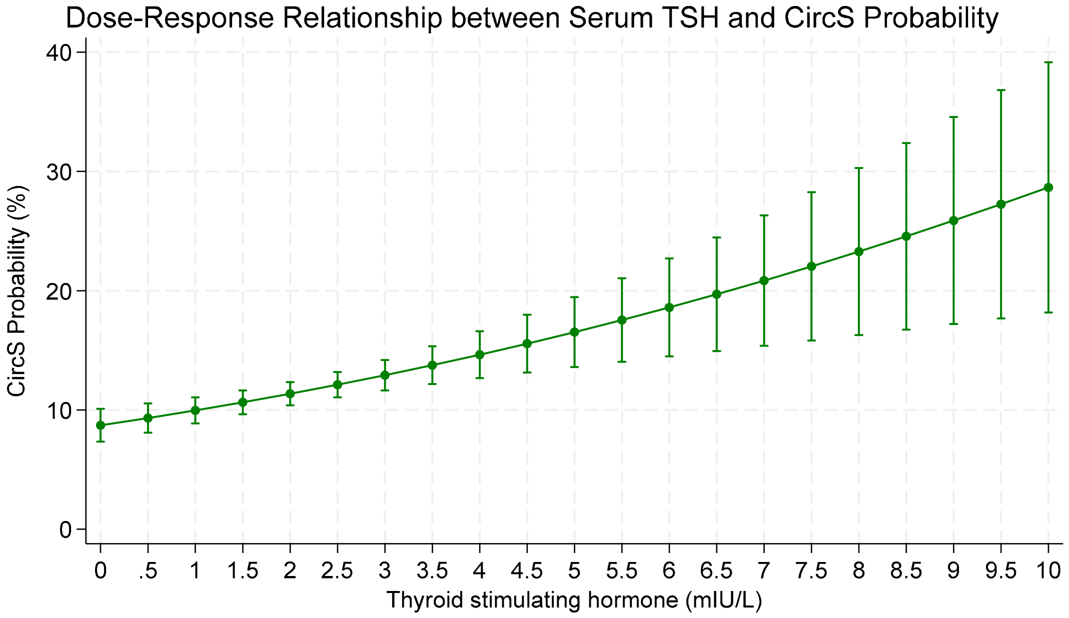

Figure S4. Dose-response Curve demonstrating the adjusted association between serum TSH and CircS probability (n= 3,817).[1]

[1] Model adjusted for age (centered at the mean value of 47.2 years), sex, race, poverty income ratio, education level, diet quality, physical activity (centered at the mean value of 65.5 MET hours per week), vitamin D status, CKD, and liver cirrhosis.
